# Retraction: Expanded alleles of the *FMR1* gene are related to unexplained recurrent miscarriages

**DOI:** 10.1042/BSR-20170856_RET

**Published:** 2019-04-23

*Bioscience Reports* (2017) **37**(6); https://doi.org/10.1042/BSR20170856

This article is being retracted from *Bioscience Reports* at the request of the Editor-in-Chief and the Editorial Board following receipt of a complaint which calls into question the validity of data included in the article. The authors have been contacted for an explanation, but have failed to respond.

